# Conservation stories from the front lines

**DOI:** 10.1371/journal.pbio.2005226

**Published:** 2018-02-05

**Authors:** Liza Gross, Annaliese Hettinger, Jonathan W. Moore, Liz Neeley

**Affiliations:** 1 Public Library of Science, San Francisco, California, United States of America; 2 University of California, Davis, Bodega Marine Laboratory, Bodega Bay, California, United States of America; 3 Earth to Ocean Research Group, Simon Fraser University, Burnaby, British Columbia, Canada; 4 The Story Collider, Washington DC, United States of America

This Editorial is part of the *Conservation Stories from the Front Lines Collection*

The stories of science are told many ways, in many places. Scientists share the ups and downs of the research process over raucous conference cocktails and long hours on the road, across lab benches and conference call lines, and around campfires after long days in the field. These stories underlie every scientific paper yet rarely appear alongside the tables and graphs. To read the often dull, sometimes tedious reports that fill the scientific record, you’d never know that science is a human endeavor, like any other, shaped by tragedy, comedy, and (mis)adventures.

In this issue of *PLOS Biology*, we highlight the deeply human side of research in a new collection, “Conservation Stories from the Front Lines.” These narratives present peer-reviewed and robust science but also include the muddy boots and bloody knees, ravaging mosquitoes, crushing disappointment, and occasional euphoria their authors experienced. We deliberately sought stories of triumphs and tragedies, successes and failures, and invited a diverse group of scientists to submit contributions written in their own voices. Rather than cling to a standard structure, we asked authors to choose their own format to best present their ideas, experiences, results, and conclusions in a style that is compelling, concise, and accessible.

Our focus in this collection is conservation—science that speaks to the management and preservation of species and ecosystems. Contributions range from perspectives on an existing body of research to the presentation of novel research findings. Authors were encouraged to breathe life into their scientific stories by incorporating narrative elements such as characters, scenes, conflict, and resolution.

Karen Lips describes the agony of watching the rainforest frogs she studied for years suddenly and mysteriously disappear [1]. Nick Haddad shares epiphanies about the recovery of rare species gleaned from humbling struggles with his health [2]. Elizabeth Hadly confesses her fear that the days when government leaders acted on evidence of human-driven planetary emergencies may be gone [3]. Emmanuel Frimpong urges us to consider how the ecological role of an overlooked fish warrants a new approach to freshwater fish conservation [4]. And Sergio Avila-Villegas reveals how a painful encounter with a jaguar changed the trajectory of his life and his life’s work [5].

Stories are powerful, even transformative. Most of us are aware of that power, based either on personal experience or on stories we know from the media and entertainment industries. But we can go beyond intuition and look to the scientific study of stories. Compared with argumentative or evidence-based communication, narratives focus on causal linkages among a sequence of events influenced by the actions of specific characters. They often carry an emotional punch and relate these events in a way that resonates with readers. As a result, narrative has the power to improve comprehension, increase topical interest, influence real-world beliefs, and achieve persuasive outcomes [6].

It is precisely because stories are so compelling that they must be considered carefully. Simple, appealing, but terribly misleading narratives can result in the rejection of empirical reality, as we see in climate change and vaccine safety discussions. Stories can be seductive, even among technical experts. It is no surprise that storytelling as a science communication form has been critiqued as manipulative and inappropriate [7,8]. We emphatically agree on the need for rigor and careful representation of reality within science narratives [9]. The Conservation Story submissions were peer reviewed to vouchsafe their empirical footing. The result is a collection of empirically robust stories in which scientists reflect on the process and results of their research to communicate with audiences in ways that traditional papers can’t.

Scientists are increasingly recognizing the need to find new ways to effectively engage with a diversity of audiences [10–14]. Here, we’ve revisited the historical version of scientific communication by turning peer-reviewed papers into evidence-based, scientific stories. We don’t know where this experiment will go—perhaps it will end with this single collection. But conceivably, it could catalyze further experiments with peer-reviewed scientific narratives. We hope it does. As we grapple with emerging crises wrought by a changing climate and plummeting biodiversity, we’ll need to explore every possible avenue for sharing the best available science with audiences far beyond the academy.
